# Unequal disease burdens: exploring the intersection of gender, health and disease dynamics in China

**DOI:** 10.1186/s12889-025-23354-3

**Published:** 2025-07-02

**Authors:** Xueneng Yang, Ruijuan Li, Junfei Liu, Jun Shu, Hanbo Chen, Minglin Dong, Jia Lv, Yong Yuan, Qiangqiang Song, Limin Guo, Ming Zeng, Bo Li

**Affiliations:** 1 Department of Traumatology, Second Affiliated Hospital of Kunming Medical University, Kunming, Yunnan China; 2 Department of Burn, Second Affiliated Hospital of Kunming Medical University, Kunming, Yunnan China; 3 Department of Geriatric Orthopedics, Affiliated Hospital of Yunnan University, Kunming, Yunnan China

**Keywords:** Gender differences, Disease burden, Disability-adjusted life years, Health trends in China, Non-communicable diseases

## Abstract

**Background:**

Gender differences have a significant impact on disease burden. Although there is extensive research on disease burden in China, the age-related and long-term trends in gender differences for major diseases are not clear. This study analyzes the gender differences in the burden of 20 major diseases in China from 1990 to 2021, revealing temporal and age-related trends, and providing evidence for gender-sensitive public health policies.

**Methods:**

This study uses data from the Global Burden of Disease Study 2021 (GBD 2021) to analyze gender differences in 20 major diseases in China. Absolute differences (male DALY − female DALY) and relative differences (absolute difference / male DALY × 100%) were calculated to analyze the long-term trends and age-related characteristics of gender differences across different age groups (0–14 years, 15–49 years, 50–69 years, 70 + years).

**Results:**

In 2021, diseases with a significantly higher burden for males included stroke (male 31,862,593.3 vs. female 21,328,097.8; absolute difference 10,534,495.5, relative difference 33.1%), cirrhosis and other chronic liver diseases (relative difference 69.3%), and lung cancer (absolute difference 6,743,543.2). Alzheimer’s disease and other dementias were the only diseases with a higher burden in females (female 6,500,198.7 vs. male 3,572,278.8; relative difference − 82%). Gender differences were present in early childhood, especially in neonatal disorders and road injuries, while stroke showed the largest gender difference in the elderly. Between 1990 and 2021, the gender difference in diabetes mellitus declined (from 8.1% lower in males to 10.3% higher in males). The gender gap continued to widen for cirrhosis (relative difference increased from 55.1 to 69.3%) and ischemic heart disease (from 19.7 to 34.3%).

**Conclusion:**

Over the past 30 years, gender differences in disease burden in China have changed dynamically, influenced by biological, behavioral, and sociocultural factors. Public health policies should focus on high-risk behaviors in males, such as smoking, drinking, and occupational exposure, by implementing cardiovascular disease and cancer screening programs. For elderly females, a community care network for dementia and early intervention for postmenopausal metabolic diseases should be established. Optimizing healthcare resource allocation and promoting targeted interventions can reduce gender health inequalities.

**Clinical trial number:**

Not applicable.

**Supplementary Information:**

The online version contains supplementary material available at 10.1186/s12889-025-23354-3.

## Introduction

Gender differences significantly influence disease burden, shaped by the interaction of biological factors, sociocultural contexts, and behavioral patterns [1]. These interconnected factors contribute to the onset, progression, and outcomes of various diseases [2]. Disease burden, a key public health metric, assesses the impact of specific diseases on population health and socioeconomic factors, typically including incidence, mortality, disability-adjusted life years (DALYs), and economic costs. Therefore, analyzing gender differences is crucial for developing effective public health strategies.

Globally, the influence of gender differences on disease burden has attracted widespread attention. For example, men bear a heavier burden of cardiovascular diseases (with global male DALYs at 6727.92 person-years, significantly higher than 4808.48 person-years in women) [3]. In contrast, women face a higher burden of chronic diseases and mental health problems, such as type 2 diabetes [4], chronic obstructive pulmonary disease [5], osteoporosis [6], arthritis [7], and depression [8]. As a populous country, China’s disease burden and gender disparities deserve special attention. While there has been extensive research on disease burden in China, such as studies on atherosclerosis [9], hepatitis B [10], and gastrointestinal diseases [11], these studies often focus on individual diseases and gender differences (e.g., which gender faces a heavier burden). They lack systematic analysis of trends in gender differences, age-related disparities, and the degree of these changes. Despite widespread attention to gender differences, the lack of analysis on the absolute values and extent of gender differences in disease burden limits our understanding of their dynamic changes, impacting the proper adjustment of public health policies and healthcare resources. Identifying gender differences in the top 20 global diseases in China will help us better understand the impact of gender on health and provide scientific evidence for developing more precise public health policies in the future.

With China’s rapid socioeconomic development [12], changes in urban-rural disparities [13], and the aging population [14], the role of gender in disease burden is also undergoing dynamic changes [15]. On one hand, there are significant differences between men and women in behaviors (such as smoking, drinking, and physical activity) and health awareness [16, 17]. On the other hand, women’s social status in terms of education level, economic independence, and access to healthcare has gradually improved [18, 19], which may be altering their health risk profile. Meanwhile, gender exposure patterns in certain chronic diseases and mental health issues have also changed. For example, men remain at high risk for chronic lung disease and cardiovascular diseases [20, 21], while women face a greater burden of depression and Alzheimer’s disease [22, 23]. Therefore, continuous monitoring and analysis of these changes will help better address China’s complex public health needs, such as population aging and unequal healthcare resource distribution, and ensure that the policies and interventions implemented remain effective. Such analyses are of great significance for improving the health of different gender groups and reducing the overall disease burden.

The Global Burden of Disease Study (GBD) is a global research project jointly initiated by the World Health Organization (WHO) and the World Bank, and implemented by the Institute for Health Metrics and Evaluation (IHME) at the University of Washington. The aim of the study is to provide key data for global health status and development [23]. GBD uses a unified modeling approach, combining data from multiple sources, to provide long-term, comparable results across dimensions such as gender, age, and geographic region. Its core indicator, DALY, is a comprehensive measure of the impact of diseases on mortality and quality of life, reflecting the loss of healthy life years due to factors like disease-related death, disability, long-term bed rest, and pain [24, 25].

This study focuses on the top 20 major diseases ranked by DALY values from the Global Burden of Disease Study 2021 (GBD 2021), covering a range of categories such as cardiovascular diseases, respiratory diseases, mental disorders, metabolic diseases, infectious diseases, and injuries (specific diseases are listed in the methods section). We systematically analyze the disease burden differences between genders for these diseases in China from 1990 to 2021, covering all age groups from infancy to old age. By comparing long-term trends across gender dimensions, the study aims to reveal the changing trends in gender differences, provide a comprehensive understanding of the gender structure of disease burden in China, identify high-risk gender-related populations across different age groups and disease types, and offer scientific support for the development of gender-sensitive public health policies and interventions.

## Methods

### Data source

The GBD database is built on internationally recognized principles of open data and global health ethics, ensuring transparency, accessibility, and scientific value. It follows the Guidelines for Accurate and Transparent Health Estimates Reporting (GATHER) principles. The data for this study were sourced from the Global Burden of Disease Study 2021 (GBD 2021), which provides comprehensive estimates of disease burden in China. The GBD 2021 database uses standardized metrics and rigorous methods to analyze trends in incidence and mortality for 371 diseases and injuries across 204 countries and territories, as well as 811 subnational regions [26]. We utilized these estimates to quantify gender differences in the burden of the top 20 diseases contributing to disability-adjusted life years (DALYs) for both males and females in China.

### Gender difference analysis

This study extracted estimates of disease burden from GBD 2021, focusing on trends in gender differences from 1990 to 2021. Due to data limitations, we were unable to differentiate the specific impacts of biological sex and social gender on disease burden. The data were primarily classified by biological sex, or in some cases, a combination of biological and social gender. Nonetheless, we recognize that both biological and social gender factors significantly influence disease burden across different stages of life [27].

### Disability-adjusted life years (DALYs)

In this study, disability-adjusted life years (DALYs) was the primary metric used to assess disease burden. DALYs is a comprehensive global health indicator that measures total health loss from both fatal and non-fatal conditions [28]. It combines years of life lost (YLL) due to premature death and years lived with disability (YLD). One DALY corresponds to the loss of one year of healthy life. Detailed methods for estimating DALYs have been described in previous studies [25, 29–31].

### Scope and geographic location

The DALY estimates from GBD 2021 apply to both males and females across 20 standardized age groups in 204 countries and territories. We selected China as the study location to analyze how its unique socioeconomic context and health challenges influence gender differences in the burden of major global diseases. To ensure broader public health relevance, gender-specific diseases (such as cervical cancer and prostate cancer) were excluded from the analysis, avoiding interference with gender difference analysis and ensuring a more accurate comparison of disease burden between males and females in non-gender-specific diseases, thus improving the scientific validity of the results.

### Timeframe and cause levels

This study analyzed data from 1990 to 2021. This time span allowed for tracking changes in disease burden among Chinese males and females at the national level, evaluating the effectiveness of public health policies and interventions, and providing historical evidence for future policy development. The study focused on the top 20 major diseases and injuries ranked under Level 3 causes in the GBD 2021 database, ensuring the broad applicability of the results. These include tuberculosis; tracheal, bronchus, and lung cancer; stroke; stomach cancer; road injuries; other COVID-19 pandemic-related outcomes; neonatal disorders; malaria; lower respiratory infections; ischemic heart disease; hypertensive heart disease; falls; diarrheal diseases; diabetes mellitus; COVID-19; colon and rectum cancer; cirrhosis and other chronic liver diseases; chronic obstructive pulmonary disease; chronic kidney disease; and Alzheimer’s disease and other dementias.

### Data analysis

We used R software to analyze the disease burden among Chinese males and females across different age groups (infants and children: 0–14 years, young adults: 15–49 years, middle-aged adults: 50–69 years, and elderly: 70 + years) and over time. Absolute differences were calculated by subtracting female DALYs from male DALYs, with positive values indicating a higher burden for males. Relative differences were calculated by dividing the absolute difference by male DALYs and multiplying by 100, with positive percentages indicating a higher relative burden for males. All calculations were based on the original data from the GBD database and included 95% uncertainty intervals (95UI). Finally, we analyzed the trends in gender differences over time and age groups, and explored the dynamic changes in disease burden by gender and age. Relevant literature supports this research methodology [30].

## Results

### Comparative analysis of global and Chinese disease burden

**Fig. 1:**
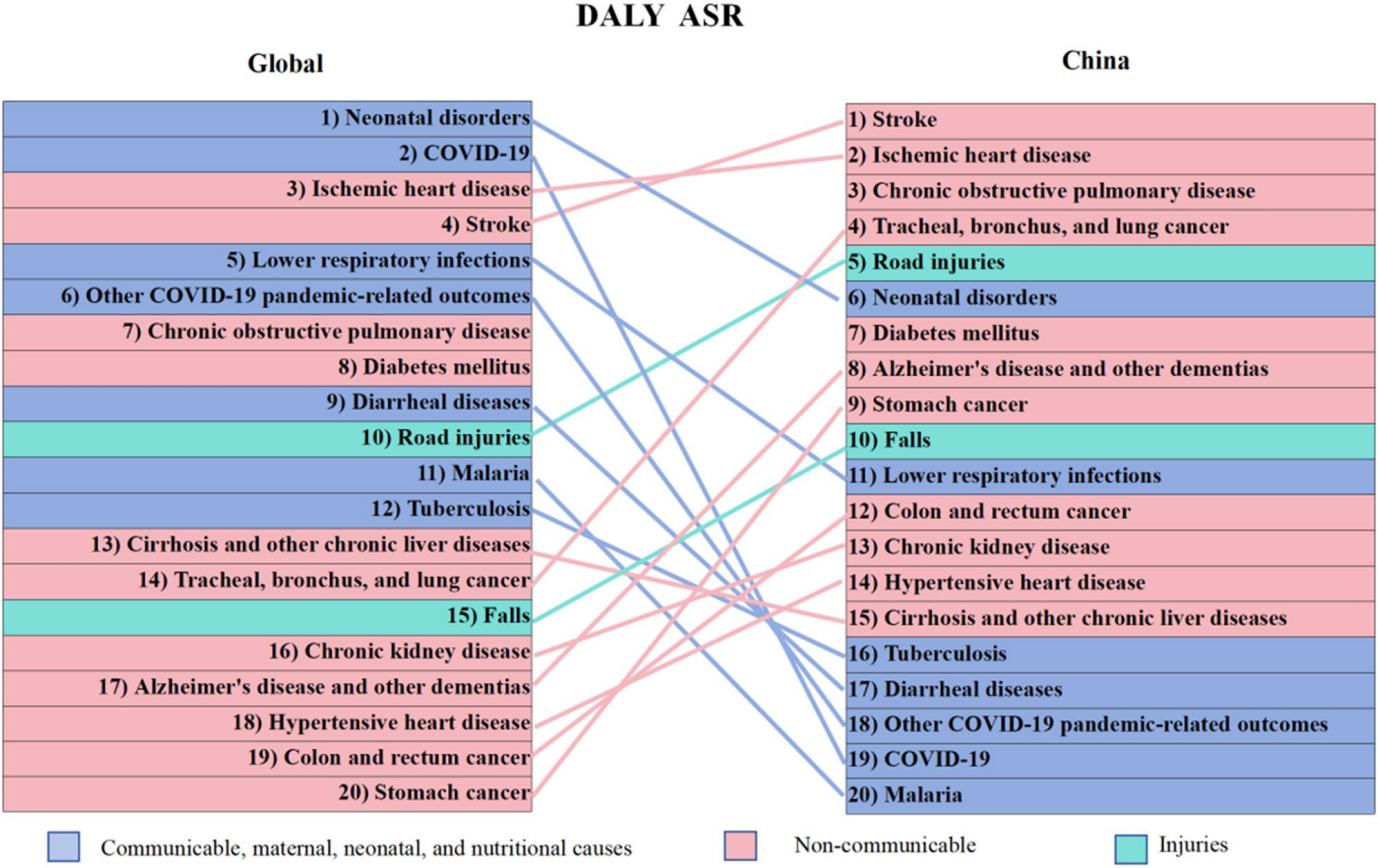
Ranking of the Top 20 Global Causes of Age-Standardized Disability-Adjusted Life Years in 2021: Global vs. China. This figure displays the 2021 rankings of the top 20 causes of age-standardized DALYs globally and in China. Dark blue represents infectious, maternal, neonatal, and nutritional diseases, red indicates non-communicable diseases, and light blue highlights injuries

In 2021, neonatal disorders were the leading cause of global disease burden, accounting for 186,366,011.8 DALYs, followed by COVID-19 and ischemic heart disease. Globally, infectious diseases, along with maternal, neonatal, and nutritional conditions, dominate the disease burden. In contrast, in China, the leading cause of disease burden in 2021 was stroke, contributing 53,190,691.2 DALYs, followed by ischemic heart disease (35,672,627.0 DALYs) and chronic obstructive pulmonary disease (23,640,321.0 DALYs). In China, the primary contributors to disease burden are predominantly non-communicable diseases. (Fig. 1)

### Gender differences in the burden of major diseases in China

**Fig. 2:**
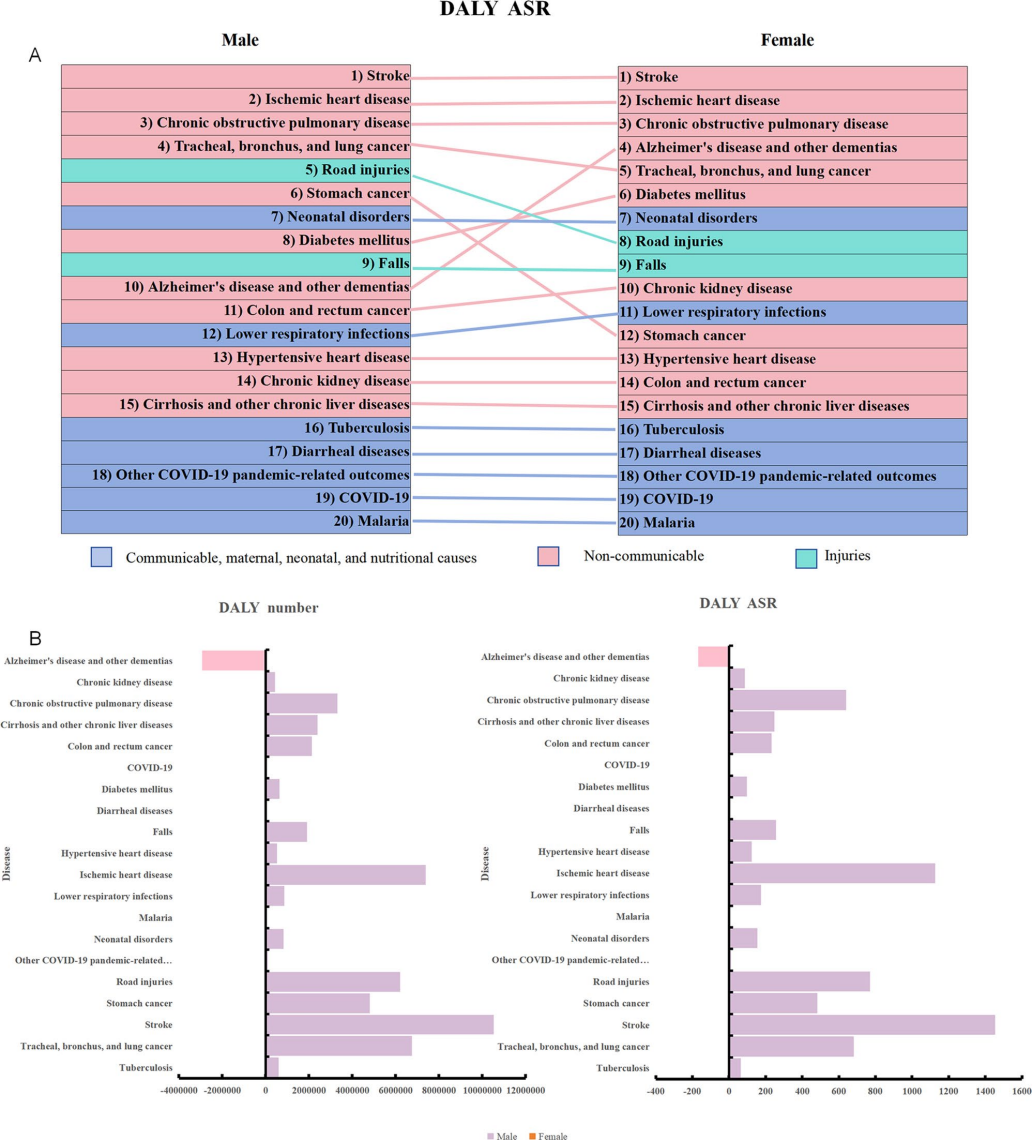
**A**. Ranking of the Top 20 Causes of Age-Standardized Disability-Adjusted Life Years in 2021 for Males and Females in China. This figure shows the 2021 rankings of the top 20 causes of age-standardized DALYs by gender in China. Dark blue represents infectious, maternal, neonatal, and nutritional diseases, red indicates non-communicable diseases, and light blue denotes injuries. **B**. Absolute Gender Differences in Age-Standardized Disability-Adjusted Life Years for the Top 20 Causes in China, 2021. This section illustrates the absolute differences between males and females, calculated as male DALYs minus female DALYs. Positive values (purple) indicate a higher burden for males, while negative values (red) indicate a higher burden for females

In 2021, the top three diseases contributing to the DALY burden for both males and females in China were stroke(31,862,593.3 vs. 21,328,097.8), ischemic heart disease(21,529,833.9 vs. 14,142,793.1), and COPD(3,281,415.2 vs. 2,846,507.5). Alzheimer’s disease and other dementias were the only conditions with a higher burden in females, showing an absolute difference of 2,927,919.9 DALYs and a relative difference of 82%. Among the diseases with a high disease burden for males, stroke showed the largest absolute difference, with 10,534,495.5 DALYs, while cirrhosis and other chronic liver diseases showed the largest relative difference at 69.3%. Compared to 1990, the gender gap narrowed only for diarrheal diseases, lower respiratory infections, neonatal disorders, and tuberculosis, while the gender difference in diabetes mellitus reversed. For all other diseases, the gender gap increased. In terms of age-standardized DALY rates, only Alzheimer’s disease and other dementias had a higher burden for females, at 167.7 per 100,000 population. The largest absolute difference was for stroke, at 1,453.9 per 100,000, while cirrhosis and other chronic liver diseases showed the greatest relative difference at 71.1%. Compared to 1990, the gender gap reversed for diabetes mellitus and diarrheal diseases, while the gap narrowed for COPD, cirrhosis, chronic liver diseases, falls, lower respiratory infections, neonatal disorders, road injuries, stomach cancer, trachea, bronchus, and lung cancer, and tuberculosis. For all other diseases, the gender gap widened. (Fig. 2. Table 1. Table 2).

**Table 1:**
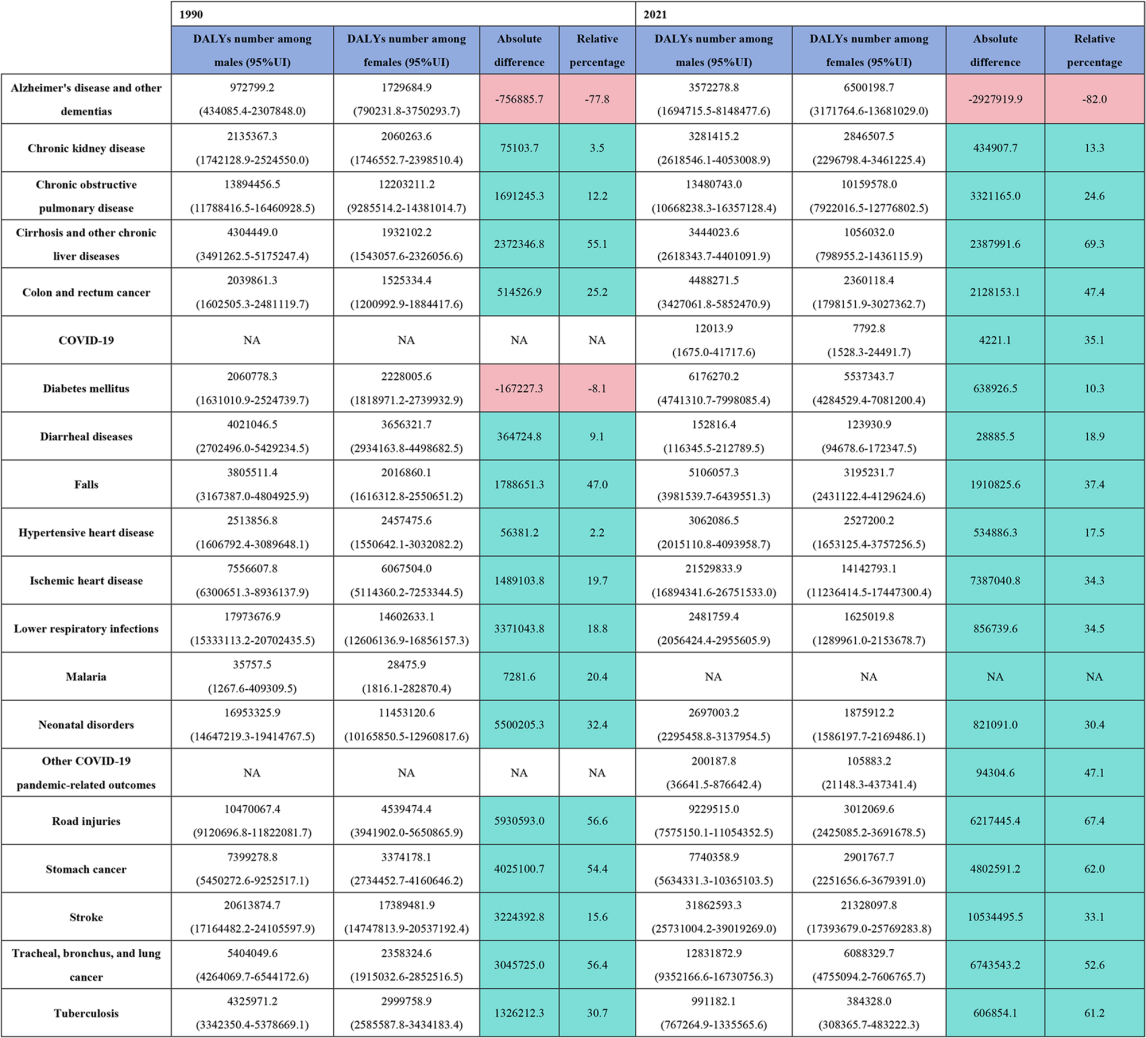
Gender differences in the burden of major diseases in china: dalys and their 95% uncertainty intervals (UI) for 1990 and 2021, including absolute and relative differences. This table presents the gender disparities in the top 20 causes of the global disease burden in China for 1990 and 2021, measured in Disability-Adjusted life years (DALYs). The absolute difference between males and females is calculated by Subtracting female dalys from male dalys, with positive values indicating a higher burden for males. The relafive difference is obtained by dividing the absolute difference by male dalys and multiplying by 100,where positive percentages indicate a greater relative burden for males. The cell color coding shows whether the absolute and relafive disparities disproportionately affect females (red) or males (light blue). dalys = Disability-Adjusted life years; UI = uncertainty interval; na = not available

**Table 2:**
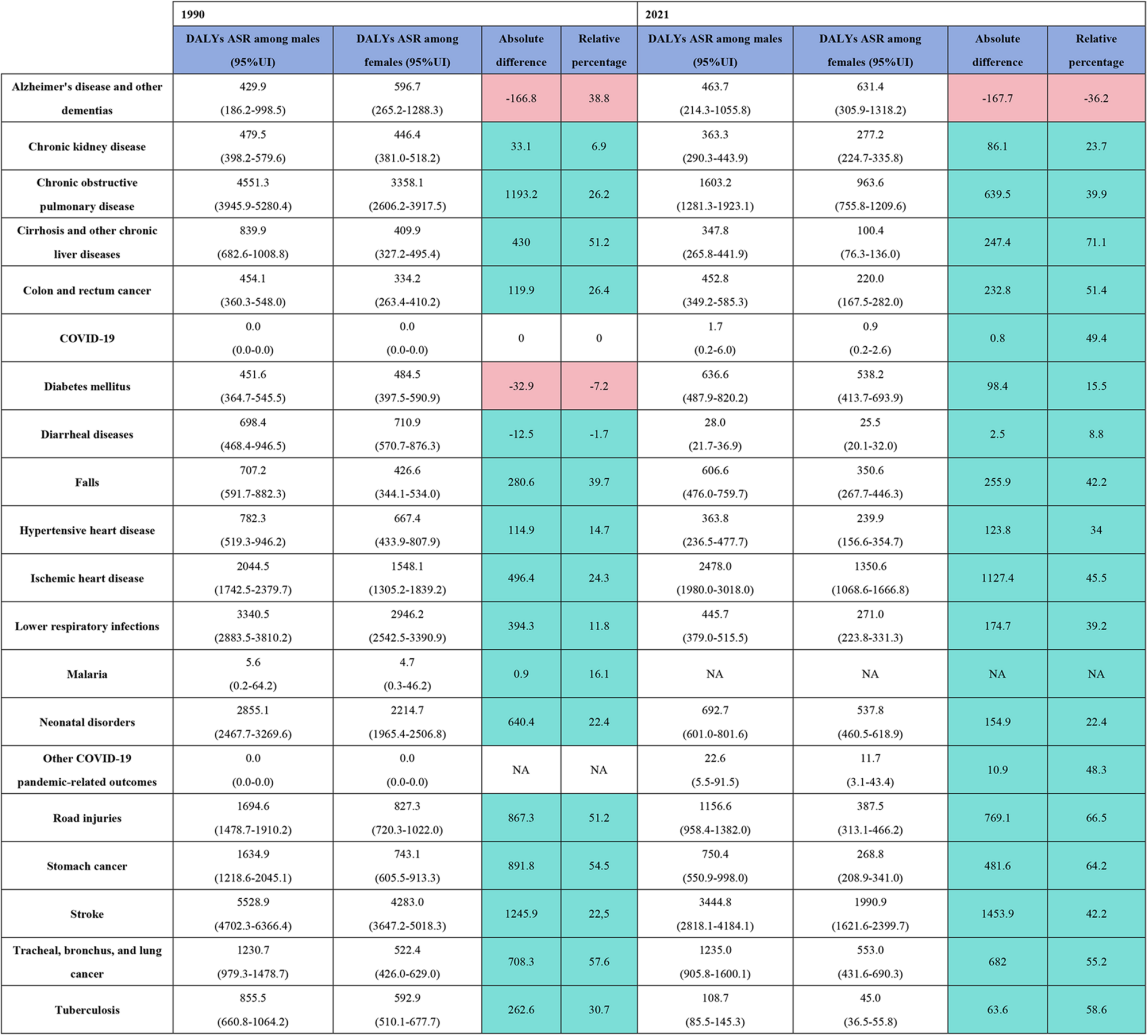
Age-Standardized Disability-Adjusted life years (ASDALYs) and their 95% uncertainty intervals (UI) for gender differences in major disease burden in china, 1990 and 2021, including absolute and relative differences. This table presents gender disparifies in the top 20 causes of disease burden in China for 1990 and 2021, measured by Age-Standardized Disability-Adjusted life years (ASDALYs). The absolute difference between males and females is calculated by Subtracting female ASDALYs from male asdalys, with positive values indicating a higher burden for males. The relafive difference is calculated by dividing the absolute difference by male ASDALYs and mulfiplying by 100, with positive percentages indicafing a greater relative burden for males. Cell color coding highlights whether the absolute and relafive disparities disproportionately affect females (red) or males (light blue). UI = uncertainty interval; na = not available

### Age-related trends in gender differences

**Fig. 3:**
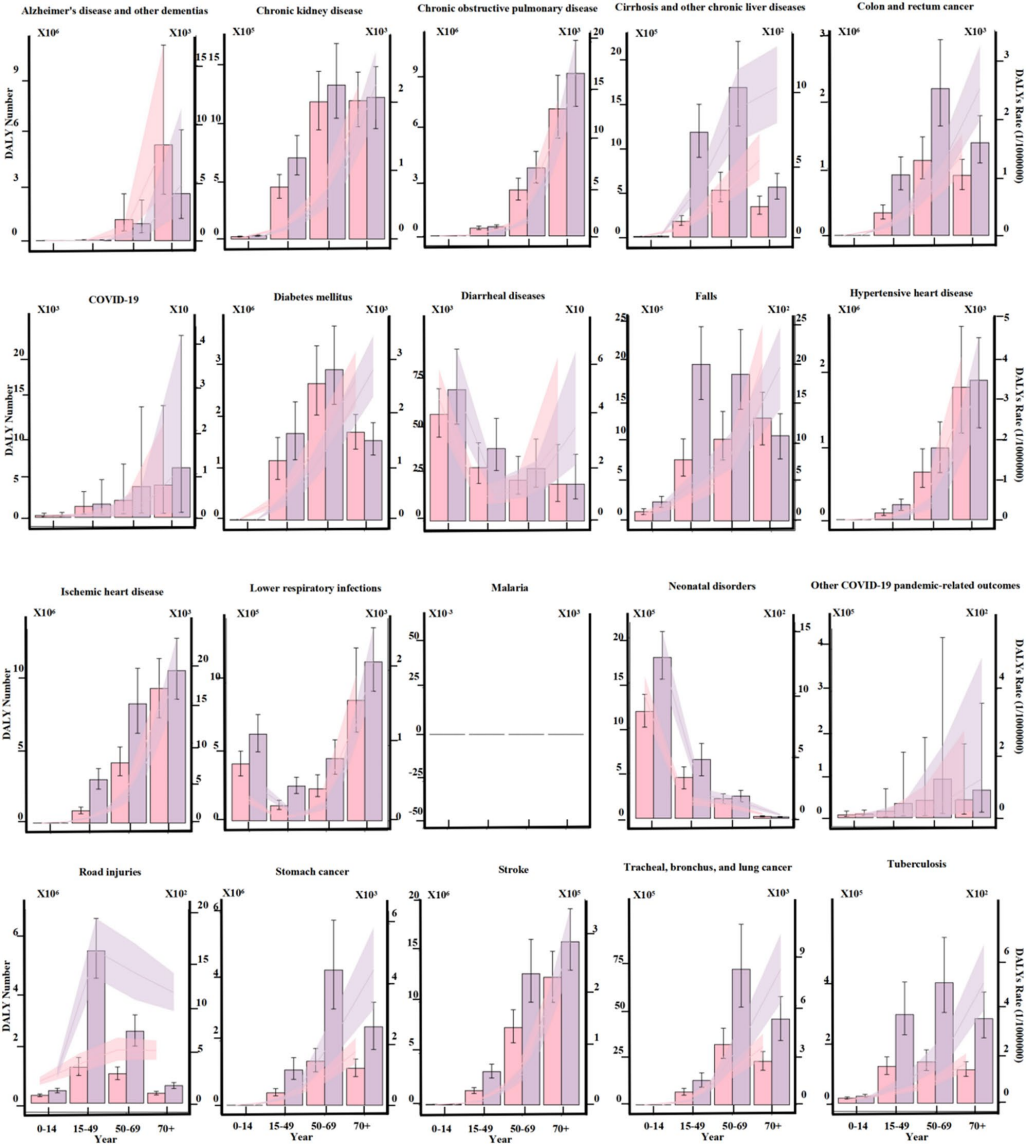
Age Trends in Disability-Adjusted Life Years (DALYs) and DALY Rates for Various Diseases in 2021. This figure illustrates the differences between males and females across different age groups for the top 20 causes in 2021, based on Disability-Adjusted Life Years (DALYs) and DALY rates. Red represents females, while purple represents males. The 95% uncertainty intervals (UI) for each estimate are depicted as shaded areas around the point estimates. The shape of the points indicates the absolute differences

Gender differences in the burden of major diseases in China follow complex, dynamic patterns across age groups. Malaria has been effectively controlled, with no disease burden observed in any age group. Except for Alzheimer’s disease and other dementias, chronic obstructive pulmonary disease, colon and rectum cancer, hypertensive heart disease, ischemic heart disease, stomach cancer, and tracheal, bronchus, and lung cancer, most diseases show gender disparities from infancy, with males generally experiencing a higher burden. Notably, for neonatal disorders, the burden for males is significantly higher than for females, reaching 598,940.17 DALYs. In young adulthood (15–49 years), the disease burden for males increases significantly, particularly for road injuries, which account for 4,224,200.343 DALYs, widening the gender gap. Meanwhile, gender differences narrow for lower respiratory infections, neonatal disorders, and diarrheal diseases, while the burden for diabetes mellitus reverses, becoming higher for females. Alzheimer’s disease and other dementias also exhibit a heavier burden for females in this age group. In middle age (50–69 years), males show a higher burden for all diseases except Alzheimer’s disease and other dementias, with stroke presenting the largest gender disparity at 5,231,559.374 DALYs. The gender gaps for chronic kidney disease, diabetes mellitus, diarrheal diseases, falls, neonatal disorders, and road injuries narrow, while the gaps for other diseases continue to widen. In the elderly group (70 + years), stroke remains the disease with the largest gender difference at 3,454,842.28 DALYs. However, females experience an increased burden from Alzheimer’s disease and other dementias, diabetes mellitus, falls, and neonatal disorders. Additionally, gender differences for Alzheimer’s disease and other dementias, COPD, COVID-19, and lower respiratory infections increase, while the gaps for road injuries and colon and rectum cancer narrow. Overall, gender differences in disease burden follow a cyclical pattern across the life course. With increasing age, the gender disparities in diabetes mellitus and neonatal disorders alternate, while the gaps for Alzheimer’s disease and other dementias, COPD, and COVID-19 steadily widen. In contrast, gender differences for diarrheal diseases gradually decrease, and other diseases exhibit an initial increase in gender disparity followed by a decline. (Fig. 3.)

### Temporal trends in gender differences of major disease burden in China

**Fig. 4:**
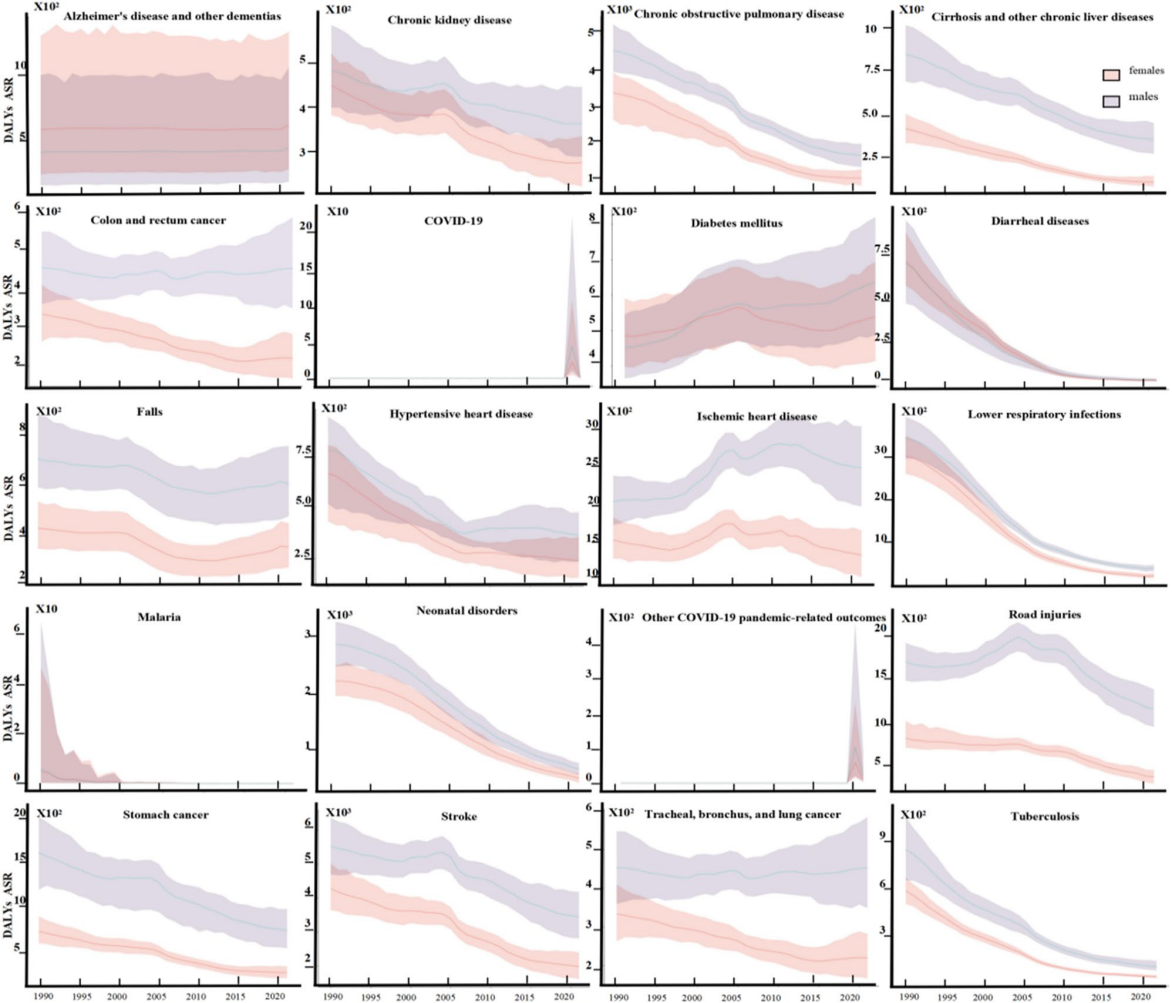
Time Trends in Age-Standardized Disability-Adjusted Life Years for Various Diseases. This figure illustrates the time trends of age-standardized Disability-Adjusted Life Years for the top 20 causes in China in 2021. The red line represents the age-standardized Disability-Adjusted Life Years for females, while the purple line represents the age-standardized Disability-Adjusted Life Years for males. The distance between the lines indicates the absolute difference between males and females

Between 1990 and 2021, gender differences in age-standardized DALY rates for major diseases in China showed diverse trends. For six diseases—Alzheimer’s disease and other dementias, chronic obstructive pulmonary disease, falls, hypertensive heart disease, lower respiratory infections, and stroke—the gender gap changed little over time. Seven diseases, including chronic kidney disease, colon and rectum cancer, ischemic heart disease, road injuries, and tracheal, bronchus, and lung cancer and so on, showed an increasing gender gap. In contrast, six diseases showed a decreasing gap, with diarrheal diseases and diabetes mellitus even reversing in gender burden. Overall, the direction of gender differences varied across diseases, reflecting a complex and dynamic pattern. (Fig. 4).

## Discussion

### Overview of gender disparities in disease burden in China

This study provides a comprehensive analysis of temporal and age-related trends in gender differences in the burden of 20 major diseases in China, based on DALYs and age-standardized DALY rates. Gender disparities in disease burden have remained significant since 1990, with males generally experiencing a higher burden than females. However, in the case of Alzheimer’s disease and other dementias, females carry a greater burden. From an age perspective, gender disparities are evident as early as infancy, with neonatal disorders and road injuries showing marked differences during childhood and adolescence, while stroke presents the largest gender gap in the elderly population. These findings demonstrate that gender differences in disease burden shift over time and across age groups. Thus, healthcare providers and policymakers should develop gender-sensitive public health strategies tailored to specific diseases to reduce gender inequalities.

### Genetic, hormonal, and immune factors driving gender differences

Gender inequality has long been a focus of extensive research, and as demonstrated in this study, significant gender differences persist in the burden of major diseases in China, influenced by biological, behavioral, and sociocultural factors. Lifelong genetic differences between males and females begin at conception when the egg is fertilized by either an X or Y sperm, establishing fundamental chromosomal differences in male and female cells [32]. Parental imprinting further contributes to these differences, with males inheriting a maternal imprint on the X chromosome, while females inherit X chromosomes from both parents, carrying both maternal and paternal imprints. These imprints can directly influence gene expression. However, some X chromosome genes escape inactivation, leading to higher expression in females, which contributes to gender susceptibility to diseases like cancer [32–34]. Additionally, the SRY gene on the Y chromosome in males triggers testicular development and a surge of testosterone during fetal development, programming gender differences in multiple organ systems and gene expression through epigenetic mechanisms. This contributes to males’ increased susceptibility to certain diseases, such as liver disease [35, 36]. Sex hormones also play a critical role in gender-related biological differences. Estrogen has anti-inflammatory properties that protect vascular endothelial and immune cells, reducing inflammation and fibrosis, thereby lowering the risk of cardiovascular diseases by improving lipid metabolism and reducing blood pressure [37, 38]. In contrast, testosterone promotes vascular remodeling, increases inflammation, and contributes to fibrosis, elevating the risk of cardiovascular disease [37–40]. This may explain the gender differences observed in cardiovascular disease burden in this study, where females consistently had a lower burden of conditions like hypertensive heart disease, ischemic heart disease, and stroke compared to males. However, this contrasts with findings in the U.S., where females have a higher cardiovascular disease burden [41], a difference that may be influenced by factors such as race, culture, and access to healthcare [42]. The impact of sex hormones is also closely related to the higher incidence and more aggressive phenotype of hepatocellular carcinoma in males. Research shows that testosterone stimulates the development of hepatocellular carcinoma, while estrogen provides a protective effect, reducing cancer occurrence. The results of this study further confirm this observation. After menopause, the decline in estrogen levels leads to metabolic disruptions, contributing to conditions such as osteoporosis and Alzheimer’s disease [43]. Studies have shown that estrogen deficiency negatively affects glucose metabolism in the brain, directly influencing the progression of Alzheimer’s disease, which may explain the higher burden of this condition in females in this study. Differences in the immune system also play a key role in gender disparities. Females generally exhibit stronger immune responses, which provide protection against certain infectious diseases. However, this heightened immune activity makes females more susceptible to autoimmune diseases, such as rheumatoid arthritis, with approximately 80% of autoimmune disease patients being female. Additionally, males exhibit a higher burden of cancer across all age groups and populations, which may be linked to biological factors. The differences in chromosome structure, where some genes on the partially inactivated X chromosome in females remain expressed, provide stronger tumor suppression. Conversely, certain genes on the male Y chromosome, such as RBMY, may contribute to malignant cell transformation [44]. Moreover, sex-specific cellular mechanisms in males may increase susceptibility to cancers such as glioblastoma. As indicated in this study, in 2021, the burden of stomach cancer, colorectal cancer, and lung cancer in males was almost 1.5 times that of females. Behavioral and lifestyle differences between genders also contribute to disparities in disease burden. Riskier behaviors, such as higher rates of smoking and alcohol consumption among males, increase their risk for diseases like heart disease and cancer [41, 45]. As demonstrated in this study, the burden of diseases such as lung cancer and liver disease has consistently been higher in males than in females. Diet and exercise also play a role in metabolic disease burden, with females generally consuming more fruits and vegetables, while males tend to eat more high-fat, high-protein foods, leading to higher rates of diabetes and fatty liver disease in males [45]. Additionally, females’ higher participation in healthy activities such as exercise may explain their lower burden of weight-related and cardiovascular diseases [46]. This may account for the study’s findings, where the disease burden for stroke and ischemic heart disease in males was nearly 1.3 times higher than in females. Sociocultural factors also significantly impact gender disparities in disease burden [42, 47–49]. Unlike biological sex, gender roles often dictate that men work in high-risk occupations such as construction, manufacturing, and mining, leading to a greater burden of chronic diseases such as lung and musculoskeletal conditions. This aligns with the study’s findings, where males had a higher burden of chronic obstructive pulmonary disease, falls, road injuries, and lung cancer. In contrast, females are more frequently involved in caregiving roles, such as nursing, education, and family care, which are associated with mental health issues like stress, depression, and anxiety. Gender roles also affect health awareness and access to healthcare resources [47–49]. Females tend to be more proactive in seeking medical attention and participating in health interventions, such as vaccinations and checkups, whereas males are less likely to seek early care, leading to poorer disease control and a higher burden later in life. Furthermore, gender bias in the healthcare system can result in delayed diagnoses for females in conditions like cardiovascular disease and an underestimation of conditions such as depression and osteoporosis in males, leading to delayed treatment and intervention [50–52]. Cultural gender imbalances in economically underdeveloped regions also limit women’s access to healthcare resources, exacerbating the burden of diseases such as depression and anemia [53].

### Age-related trends in gender differences in disease burden

Previous studies have only briefly addressed the age-related trends in gender differences [54], and the age trends in gender disparities for major diseases in China remain unclear. The findings of this study indicate that for most major diseases in China, gender differences in disease burden tend to increase initially and then decrease with age. However, Alzheimer’s disease and other dementias, Chronic obstructive pulmonary disease, and COVID-19 show a continuous increase in gender disparity, while Diarrheal diseases exhibit a gradual decline. The cumulative effects of sex hormone levels and risk behaviors play a crucial role in these trends [55]. Before menopause, the protective effects of estrogen result in a lower disease burden for females. In contrast, males, due to gender-related high-risk behaviors, tend to experience a higher burden in middle age, leading to a gradual increase in gender differences in disease burden from infancy onwards. In old age, as hormone levels decline, females face increased risks of cardiovascular and metabolic diseases, leading to a reduction in gender disparities in disease burden. Additionally, the higher disease burden in middle-aged males (e.g., cardiovascular diseases and cancers) contributes to higher premature mortality, leaving fewer but relatively healthier males in the elderly population. In contrast, females, with longer life expectancy, are more likely to accumulate chronic diseases in later life, such as Alzheimer’s disease, osteoporosis, and other degenerative conditions [55]. This explains the increase in disease burden among elderly females and the narrowing of gender differences. Recent research also suggests that changes in gender roles contribute to the variation in gender differences with age. As men age, their gender role perceptions tend to modernize, while women’s perceptions become more traditional. However, after entering middle age, the views of both genders begin to converge, displaying a “scissors gap” trend. This aligns with the findings of this study, where most diseases show an increase in gender differences, followed by a decrease. Notably, this study also found that the gender disparities in the burden of Alzheimer’s disease and other dementias and COVID-19 continue to widen with age. The increasing gender disparity in Alzheimer’s disease and other dementias may be related to females’ longer life expectancy globally (age being a key risk factor for Alzheimer’s disease). Furthermore, the APOE-ε4 gene, a critical factor in Alzheimer’s disease, is often more highly expressed in females, making them more susceptible [56, 57]. In the case of COVID-19, the widening gender disparity could be due to females experiencing lower severity and mortality rates.

### Impact of socioeconomic development and health policies on gender disparities

The shifts in gender disparities in the burden of major diseases in China from 1990 to 2021 have been influenced by socioeconomic development, health policies, lifestyle changes, and evolving gender roles. This study shows that gender differences in age-standardized DALY rates for Alzheimer’s disease and other dementias, chronic obstructive pulmonary disease (COPD), diarrheal diseases, falls, hypertensive heart disease, lower respiratory infections, and stroke have remained relatively stable. This may be due to consistent biological factors such as physiological structure, immune function, and aging processes, as well as stable social factors like smoking and occupational dust exposure [58]. These conditions are closely associated with age, blood pressure management, and workplace hazards. In contrast, the gender gaps in age-standardized DALY rates for chronic kidney disease, colon and rectum cancer, ischemic heart disease, road injuries, and tracheal, bronchus, and lung cancer have widened. This trend can be attributed to more prevalent unhealthy behaviors among males, such as high-fat diets, decreased physical activity, smoking, and higher-risk occupational exposure, while females have seen a relative decline in these burdens [59, 60]. Additionally, the narrowing of gender differences in some diseases is closely tied to improvements in women’s socioeconomic status, better access to healthcare, and healthier lifestyle choices in China. Notably, diarrheal diseases and diabetes mellitus have shown a reversal in gender disparities. The reversal in diarrheal diseases is largely due to improved sanitation, widespread health education, and reduced exposure of women to caregiving roles. The reversal in diabetes mellitus is linked to rising obesity rates among men, deteriorating lifestyles, and increased health awareness among women. Overall, the growing burden of cardiovascular diseases and diabetes in men reflects a relative lack of public health interventions targeting males, while women have benefited more from prevention and management efforts for certain chronic diseases.

### Policy recommendations for addressing gender disparities in disease burden

In response to the changing gender disparities in major diseases from 1990 to 2021, policymakers and healthcare practitioners should prioritize interventions that target high-risk behaviors in males, particularly in the areas of cardiovascular disease, diabetes, and cancer. Promoting health education, increasing awareness of disease prevention and management, and encouraging healthier lifestyles are essential strategies [61, 62]. Regular health screenings, especially for middle-aged and older men, should be emphasized to enable early detection and intervention, reducing the burden of high-risk behaviors. Additionally, efforts should focus on minimizing occupational hazards for men in high-risk jobs by implementing stronger preventive measures, improving working conditions, and enhancing safety standards, particularly in industries such as manufacturing, construction, and mining. As the population ages, greater attention should be given to the burden of chronic diseases and mental health issues among women. Strengthening mental health support, providing long-term care services, and offering more comprehensive screening programs, especially for cardiovascular diseases and Alzheimer’s disease in middle-aged and older women, are crucial [63]. Moreover, it is vital for the government to promote equitable healthcare resource distribution and reduce disparities in access between urban and rural areas, ensuring that women in economically disadvantaged regions have the same access to quality health screenings and chronic disease management services. Policymakers should also consider the evolving gender differences across age groups and disease types, actively promoting personalized disease management strategies. Precision interventions that focus on specific genders and diseases, alongside gender-sensitive health policies, are critical for addressing the diverse health needs of both genders, ultimately advancing health equity in society.

## Limitations

This study provides a comprehensive analysis of gender differences in the burden of major diseases in China from 1990 to 2021. However, several limitations should be acknowledged. First, the analysis focuses on biological sex without fully addressing the impact of social gender factors, potentially underestimating the influence of cultural, occupational, and behavioral factors on disease burden. Second, the data were derived from the GBD 2021 database, which, while employing rigorous statistical methods, may have limitations in data completeness and accuracy for certain diseases or populations. Additionally, the study limits its scope to the top 20 diseases, potentially overlooking other conditions with lower disease burden but significant health implications. Importantly, the GBD framework is based on a single-disease model and does not adequately reflect the combined effects of multimorbidity. In real-world settings, individuals often suffer from two or more chronic conditions simultaneously, and the patterns of multimorbidity can differ substantially between genders, potentially leading to underestimation or misrepresentation of the actual burden. The cross-sectional design also precludes a detailed understanding of the dynamic changes in disease burden over time or the long-term effects of health interventions. Finally, the study primarily uses DALYs as the metric, which, while comprehensive, may not fully capture the socioeconomic or psychological impact of specific diseases. Future research should incorporate social gender dimensions, expand the range of diseases analyzed, consider the role of multimorbidity, and employ longitudinal study designs to gain a more holistic understanding of gender disparities in disease burden and inform more inclusive and targeted public health policies.

## Conclusion

This study systematically examined gender differences in the burden of 20 major diseases in China from 1990 to 2021, highlighting key trends across different age groups. The findings show that men bear a significantly higher burden of cardiovascular diseases, diabetes, and cancers, while women carry a greater burden of chronic conditions such as Alzheimer’s disease. With increasing age, many diseases exhibit an initial widening of gender disparities, followed by a narrowing trend, while some conditions show the opposite pattern. These gender differences are likely influenced by a combination of biological factors, behavioral patterns, sociocultural contexts, and health policies. To effectively address these disparities, gender considerations should be integrated into public health policies and interventions, particularly those targeting high-risk behaviors and gender-specific health management strategies. Furthermore, future research should aim to refine gender categorization and reduce data biases to better understand the complex role of gender in disease burden, thereby enabling more precise health management and promoting health equity.

## Electronic supplementary material

Below is the link to the electronic supplementary material.

Supplementary Material 1

## Data Availability

The relevant data from the literature is stored in the attachments. For further inquiries, please feel free to contact the author. Additionally, the data can be accessed directly via the following link: https://www.healthdata.org/research-analysis/gbd.
